# The international survey on the management of allergic rhinitis by physicians and patients (ISMAR)

**DOI:** 10.1186/s40413-015-0057-0

**Published:** 2015-03-20

**Authors:** Carlos E Baena-Cagnani, Giorgio W Canonica, Mohamed Zaky Helal, René Maximiliano Gómez, Enrico Compalati, Mario E Zernotti, Mario Sanchez-Borges, Fabio F Morato Castro, Margarita Murrieta Aguttes, Aida López-Garcia, Faheem A Tadros, ISMAR Study Group

**Affiliations:** Research in Respiratory Medicine, Faculty of Medicine, Catholic University of Cordoba - Argentina, Santa Rosa 381, X 5000 ESG Córdoba, Argentina; Respiratory Diseases & Allergy, University of Genoa IRCCS AOU San Martino-IST, Genoa, Italy; Oto-Rhino-Laryngology, Faculty of Medicine, Ain Shams University, Ahmed Lotfy Al-Sayed Street Abbassia, Cairo, 11341 Egypt; Ayre Foundation, Head, Research & Education, Piedrabuena, 702 (A4400DSD) Salta Argentina; Universidad Católica de Córdoba, Córdoba, Argentina; Clinical Immunology, Faculty of Medicine, Central University of Venezuela, Clinica El Avila 6a Transversal de Urbanización Altamira, Caracas, 1060 Venezuela; Universidade de São Paulo, Faculdade de Medicina da Universidade de São Paulo, 24 Departamento de Clínica Médica. Av. Dr. Enéas de Carvalho Aguiar, 155 Cerqueira Cesar, Sao Paulo, SP Brasil; Medical Director Consumer Health Care Division, Allergy Department, Sanofi, 9, Avenue Romain Rolland, Paris, 75014 France; Pediatric Allergy & Immunology Department, Hospital Universitario de Puebla, 25 poniente y 13 sur Centro 3, Puebla, CP 72220 México; ENT Consultant, Al Zahra Hospital, P O Box 3499, Sharjah, United Arab Emirates

## Abstract

Epidemiologic studies suggest that the prevalence of allergic rhinitis (AR) is rising worldwide. Several reports, in fact, indicate increasing trends in the prevalence of AR especially in developing countries, likely related to the environment and climate changes and the adoption of an urbanized Western lifestyle. The primary objective of the present study was to collect information about management in real-life settings, including a characterization of typical patients’ profile referring to physicians, the disease features, the common approaches to diagnostic assessments and therapeutic decisions. This was an international, multicenter, cross-sectional study conducted in adults or children (≥6 years) suffering from rhinitis confirmed by physician's diagnosis for at least one year. The 234 physicians who participated in the study included a total of 2778 patients in Egypt, Mexico, Brazil, Colombia, Guatemala, Iran, Venezuela, Argentina, Israel, Kuwait and United Arab Emirates. It was found that clinical history was the selected tool to diagnose and categorize AR patients (97.1%), with less than half of patients undergoing allergy testing, may be explaining the scarce use of immunotherapy on management of disease. Out of 2776 patients, 93.4% had somehow received a recommendation to avoid allergens and irritant agent exposure. Notably, 91.4% were receiving at least one treatment at the time of the survey, mostly oral antihistamines (79.7%) and intranasal corticosteroids (66.3%). Oral antihistamines, intranasal steroids and decongestants were considered both safe and effective by patients and physicians, preferring oral and nasal route of administration. The ISMAR registry was designed according to the most accepted epidemiological recommendations, and provides interesting information regarding the management of rhinitis from a patient and physician points of view, with many similarities between the participating countries. Further efforts are required to better manage AR and its comorbidities.

**Electronic supplementary material:**

The online version of this article (doi:10.1186/s40413-015-0057-0) contains supplementary material, which is available to authorized users.

## Background

Epidemiologic studies suggest that the prevalence of allergic rhinitis (AR) is rising worldwide; 400 million of people suffer from rhinitis [1].

The cause of this increase is unknown, although some contributing factors include high concentrations of airborne allergens and pollution, less ventilation indoors, dietary factors, smoking and more sedentary lifestyles, among others. Several reports indicate trends in AR prevalence especially in developing countries, likely related to the environment and climate changes and the adoption of an urbanized Western lifestyle [2].

SIDRIA (Italian Studies on Respiratory Disorders in Children and the Environment) studies designed to fill the gap in knowledge regarding time trends of prevalence of asthma and allergic rhinitis in Italy indicated no changes in the prevalence rates of wheeze and increase in those of rhinitis and eczema among Italian children. The results of this study support the view that profound modifications in the epidemiological features of asthma and allergic diseases are occurring worldwide requiring comprehensive, continuous, epidemiologic monitoring [3].

AR is widely recognized as a public health concern. A recent survey remarked the direct impact of disease on social life, including mood changes, anxiety, depression, and impairment of cognitive function and quality of life [4].

Compared to the general population, people with AR complain more about sleep disturbance difficulty getting to sleep, waking up during the night as a result of their nasal symptoms. The increased risks of obstructive sleep apnea and resulting daytime fatigue have repercussion on working and school performances. It was estimated that in the USA about 3 million working days and 2 million school days lost per year are ascribable to AR, with estimated direct costs of between 2.1 and 5.9 billion US dollars per year [4,5,6]. Estimated productivity drops by an average of 20% on days when nasal symptoms are at their worst [4].

In a study conducted in Denmark the total annual treatment cost of AR is calculated to range between 2,784 and 16,408 DKK per patient [7].

The substantial burden of AR has prompted the international scientific community to develop international guidelines aimed at improving the disease management. ARIA (Allergic Rhinitis and its Impact on Asthma), for instance, is an evidence-based guideline developed in collaboration with the WHO for physicians and healthcare providers, stressing the well characterized links between asthma and rhinitis and providing guidance for their prevention and treatment [8].

Although guidelines provide recommendations about the best management options for most patients in most situations, morbidity of rhinitis is still high and the goal of the treatment is frequently far from being reached. This is likely due to difficulties in the phase of guidelines implementation, influenced by the characteristics of guidelines themselves, social, organizational, economic and political context and the implementation strategies [9]. The under-appreciation of the diseases represents another barrier [8,10]. Being often considered trivial, AR results under-diagnosed and under-treated as a consequence of the fact that only a small proportion of patients visit a specialist [10].

Recent surveys show that patients with AR are not satisfied with their current treatment and this may be a reason of the frequent non-adherence to therapy [9,11,12]. Despite the vast availability of treatment options, 60% of patients are “very interested” in finding a new medication and 25% are “constantly” trying different medications to find one that “works”. Some patients feel their healthcare provider does not understand their allergy treatment needs and does not take their allergy symptoms seriously [11].

The primary objective of the ISMAR study was to collect information about management in real-life settings, including a characterization of typical patients’ profile referring to physicians, the disease features, the common approaches to diagnostic assessments and therapeutic decisions. Moreover, the study was intended to draw a snapshot of the national and local peculiarities of this management and to estimate the relative prevalence of each type of rhinitis in each participating country.

## Materials and methods

### Study design and population

This was an international, multicenter, cross-sectional study conducted in children (≥6 years) and adults suffering from rhinitis confirmed by physician’s diagnosis for at least one year.

As this was a non-interventional study no allergen skin tests were required during the cross-sectional visit, albeit available information from patients’ records was recorded.

A sample of physicians was selected at random from a master list provided by each country to enter the survey. They included at least 4 physicians (2 private and 2 public practitioners) for each of the following specialties: GPs/family practitioners/internists, allergists/pulmonologists, pediatricians and ENT specialists.

Each physician was allowed to have 10 (and not more than 15) patients participating in the study. To enter the study they should have fulfilled the following inclusion criteria: male or female, adults or children (≥6 years), with rhinitis diagnosed by a physician at least one year before the survey; outpatients visiting their physician for whatever reason, with referred existence of nasal symptoms at any time. Patients participating in any clinical trial or with inability to complete the questionnaire were excluded. The number of interviewed physicians was determined to guarantee statistical significance but, globally and by country, a minimum of 200 patients by country or region had to be included. A maximum number of centers was not defined in the protocol. 234 centers were included in the study in 11 countries, 20 in Argentina, 27 in Brazil, 18 in Colombia, 47 in Egypt, 16 in Guatemala, 17 in Israel, 15 in Iran, 10 in Kuwait, 36 in Mexico, 19 in Venezuela and, 9 in the United Arab Emirates (UAE). Each selected Investigator has to include consecutive patients who meet inclusion criteria.

The study data collection was performed during one single visit.

Data were obtained through 3 types of documents: “Investigator’s Questionnaire”, “Case Report Form” and “Patient’s Questionnaire”.

In the Investigator’s Questionnaire the following data were recorded: the physician profile (age, gender, specialty, years of practicing, center location, main workplace), the number of patients with rhinitis seen per week (percentage of these patients with asthma), how they conducted AR diagnosis (key symptoms for diagnosis, complementary methods for diagnosis, severity criteria, categorization of patients), treatment prescribed, knowledge of ARIA, GINA and other guidelines, patient compliance, personal evaluation of efficacy and safety of main rhinitis treatments, evaluation of patient Quality of Life (if any).

In the Case Report Form the following were recorded: visit date, informed consent, inclusion/exclusion criteria, demographics (date of birth, gender, ethnicity), physical examination data (height, weight), smoking status, relevant medical history, data on the rhinitis diagnosis (year(s) since diagnosis, key symptoms, complementary methods, symptoms and management of rhinitis, co-morbidities [conjunctivitis, sinusitis, otitis media, nasal polyps, asthma (including hospitalization for asthma)].

In the Patient’s Questionnaire, the following data were collected: patient profile (age, gender, current occupation, location), years since diagnosis of rhinitis, smoking status, type of rhinitis, associated symptoms/pathologies, complementary methods for diagnosis, allergens/factors inducing nasal symptoms, preferred drug for nasal symptoms treatment, attitude to prescription, prescription compliance, preferred administration route, and information on patient’s education about rhinitis.

All procedures were performed in accordance with the ethical principles of the Helsinki declaration, with Good Epidemiological Practice guidelines and with the national regulations in force including data protection. The study was approved by the Ethics Committees in accordance with local regulations. Informed consent was obtained prior to any study procedure in order to use patients’ responses in public in an anonymous and confidential way.

## Results

### Physician population

The 234 physicians who participated in the study had a median age of 49 years, were mainly males with an average experience of 20 years of clinical practice. Demographic characteristics are summarized in Table 1. The most frequent specialty was allergy/pulmonology (35.9%), followed by ENT (30.3%), family practice/internal medicine (22.2%), pediatrics (11.1%). Most of them were private professionals only (41.9%) or with a mixed activity in private and public setting (41.5%); 16.7% was under full regimen of public health system. They were mainly resident in urban area (88.2%) and included a total of 2778 patients.

**Table 1:** **Physicians’ demographics and characteristics**

	**Physicians (234)**
Age, median (range)	49 (28–69)
Gender, male n (%)	180 (76.9)
Years in practice, median (range)	20 (1–41)
Place of residence, n (%)	
Urban	230 (98.7)
Suburban	0 (0)
Rural	3 (1.3)
Specialty, n (%)	
GP/internist	52 (22.2)
Allergist/pneumologist	84 (35.9)
Pediatrician	26 (11.1)
ENT	71 (30.3)
Other	1 (0.4)
Activity setting, n (%)	
Public	39 (16.7)
Private	98 (41.9)
Mixed	97 (41.5)
Working regimen, Median (range)	
No. of patients with rhinitis/week	20 (1–180)
No. of patients with asthma among patients with rhinitis	7 (0–90)

### Data on physicians’ practice

Concerning the regimen of activity, in average 20 patients with rhinitis and 7 with associated asthma were seen weekly.

**Figure 1:**
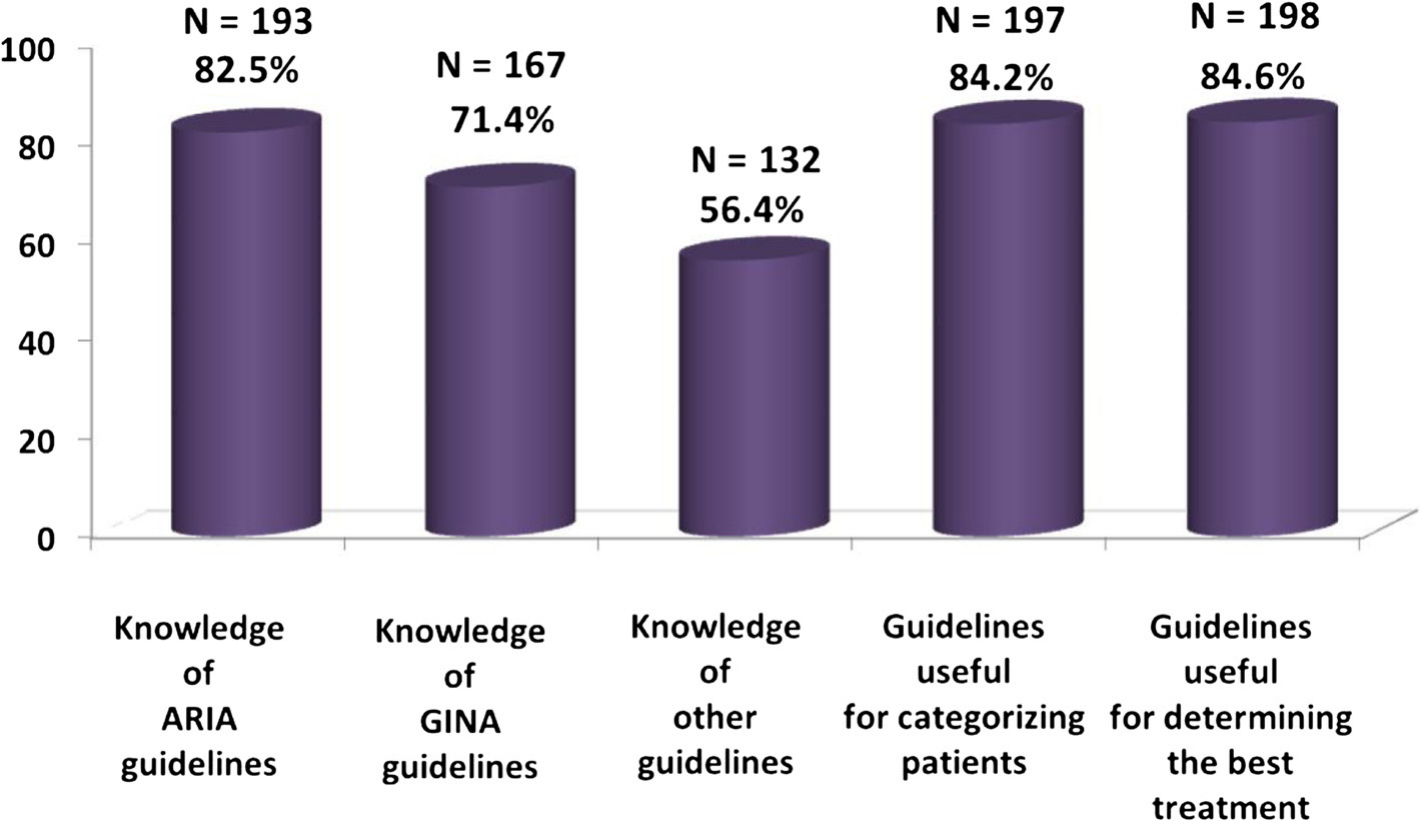
**Use of guidelines reported by physicians [Number and percentage of physicians].** Physicians answered to the following question: Do you know ARIA, GINA or other guidelines? Do you find guidelines are useful in categorizing patients? Are guidelines useful to find the best treatment for your patients?

Most physicians were aware of the existence of international guidelines; 82.5% and 71.4% of them acknowledged ARIA and GINA, respectively, but 56.4% knew also other guidelines, overall recognizing their relevance for categorizing patients in 84.2% and for determining the optimal treatment in 84.6% of cases (Figure 1).

**Figure 2:**
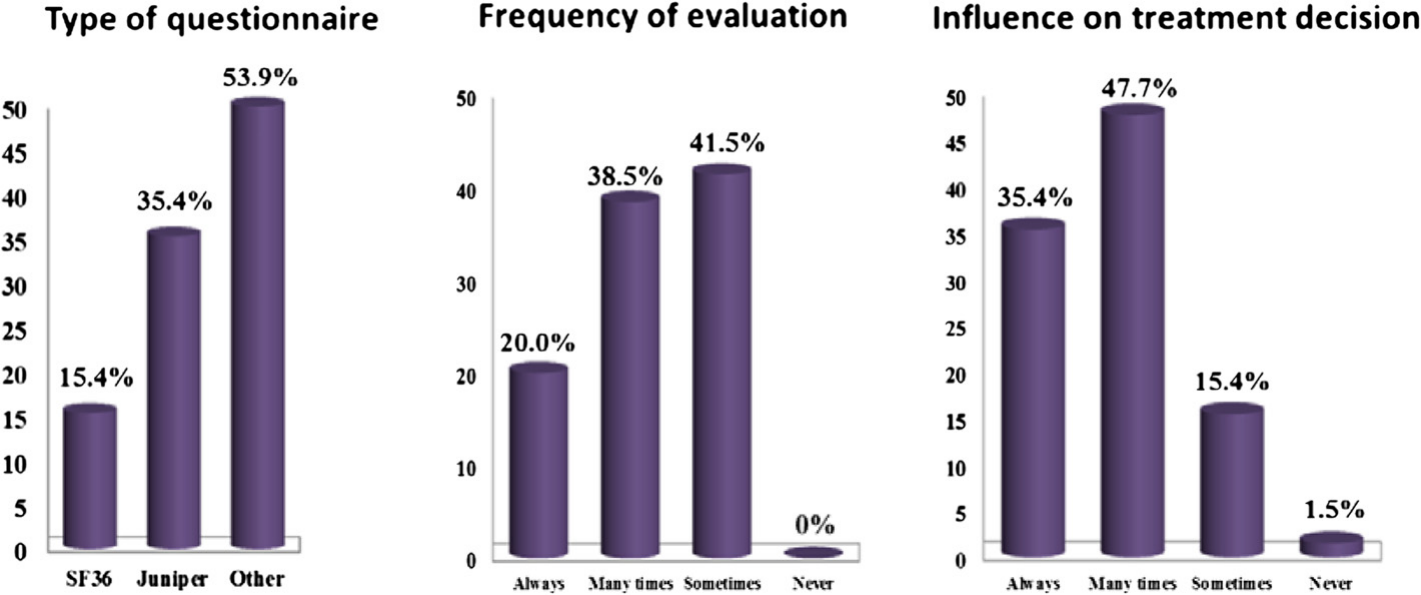
**Use of quality of life questionnaires [Percentage of physicians].** Physicians answered to the following question: Do you use standardized Quality of Life questionnaire in your clinical practice?

In this study the use of any quality of life (QoL) questionnaire was not a common practice (27.8%). Physicians reported to use the Juniper (35.4%) and the SF-36 (15.4%) questionnaires. Some physicians reported to use them frequently (20%), recurrently (38.5%), or sometimes (41.5%). Their usefulness on treatment decision was judged positively “many times”, “sometimes” or “always” by 47.7%, 15.4% and 35.4% of physicians, respectively (Figure 2).

Rhinitis has been shown to affect the quality of sleep. We observed that a high percentage of physicians (89.7%) usually assessed this outcome, mainly through a clinical history (97.1%). Polysomnography, the Epworth somnolence scale, or other tools were also used by 24.8%, 11.9% and 7.6% of physicians.

The majority of physicians (94.4%) categorized patients according to the clinical history. They preferably adopted a classification based on the severity (80.8%) and the seasonal occurrence of clinical symptoms (59.8%), however 31.2% of them used other criteria based on complementary methods. The assessment of symptoms severity was mainly based on the clinical history (98%). Although some doctors used also QoL questionnaires (38.5%), visual analogue scales (14%), numeric scales or other instruments (11%).

The most frequent reasons leading to a treatment prescription were related to the severity of symptoms (97.9%), followed by trust in clinical efficacy and safety of drugs (85.9% and 76.5%, respectively). Other reasons, ranging from 65% to 47.9% included personal experience, cost, and frequency of drug intakes, administration route, patients’ requests and categorization.

As suggested by guidelines, oral antihistamines and nasal corticosteroids were the most frequently prescribed drugs. Next in the list were oral decongestants, combinations of antihistamines and decongestants, leukotriene receptor antagonists, whereas topical antihistamines and decongestants, oral and intramuscular steroids, combinations of antihistamines and steroids were the least used drugs. Topical cromones, anticholinergic drugs, allergen-specific immunotherapy and alternative medicines were almost not used (Table 2).

**Table 2:** **Treatment characteristics and scores**

**Treatment (score)**			
**Rhinitis treatment frequency reported by physicians, median (0 = not used; 5 = most used)**	Oral anti-histamines (5)	Intranasal corticosteroids (5)	
	Oral decongestants (2)	Antihistamines + decongestants (2)	
	Leukotriene antagonists (2)	Topical antihistamines (1)	
	Intranasal decongestants (1)	Oral corticosteroids (1)	
	Intramuscular corticosteroids (1)	Antihistamines + steroids ( 1)	
	Nasal or ocular cromones (0)	Anticholinergic drugs (0)	
	Allergen s.c. immunotherapy (0)	Allergen s.c. immunotherapy (0)	
	Other immunotherapy (0)	Alternative medicines (0)	
**Treatment evaluation by physicians, median (1 = not effective; totally unsafe; 5 = extremely effective; totally safe)**		**Efficacy**	**Safety**
	Oral antihistamines	4	5
	Intranasal corticosteroids	5	4.5
	Oral decongestants	3	3
	Antihistamines + decongestants	3	3
	Leukotriene antagonists	3	4
	Topical antihistamines	1	3
	Intranasal decongestants	3	2
	Oral corticosteroids	4	2
	Intramuscular corticosteroids	3	2
	Antihistamines + steroids	3	2
**Judgments about compliance, N (%)**		**Patient (2776)**	**Physician (234)**
	Excellent	866 (31.2)	18 (7.7)
	Very good	825 (29.7)	87 (37.2)
	Good	633 (22.8)	98 (41.9)
	Intermediate	297 (10.7)	25 (10.7)
	Very poor	25 (1)	0 (0.0)
	Poor	95 (3.4)	6 (2.6)
	Negative	20 (0.7)	0 (0.0)
	Unknown	15 (0.5)	0 (0.0)
**Factors affecting treatment compliance N (%) (AEs = adverse events)**	AEs produced by medications	541 (19.5)	76 (32.5)
	Fear of AEs reported	503 (18.1)	107 (45.7)
	Route of administration	537 (19.3)	59 (25.2)
	Frequency of doses	935 (33.7)	91 (38.9)
	Efficacy of ongoing treatment	689 (24.8)	84 (35.9)
	Cost of medication	895 (32.2)	165 (70.5)
	Taste	336 (12.1)	-
	Others	399 (14.4)	-
**Patients who received educational information** ** N (%)**	Written indication	1426 (51.4)	
	Oral explanations about disease	2364 (85.2)	
	Treatment	2236 (80.6)	
	Medication side effects	1614 (58.1)	
	Other aspects	586 (21.1)	

s.c.:subcutaneous.

As expected, intranasal corticosteroids (INCs) were reported by physicians as the most effective drugs (extremely effective); while oral antihistamines were considered as the most well tolerated drugs (totally safe). On the other hand, INCs were considered safe and a-H_1_ as effective as oral corticosteroids. Oral decongestants alone or combined with antihistamines, anti-leukotrienes, intranasal decongestants, intramuscular steroids, and combinations of antihistamines plus steroid were considered equally effective. A similar safety profile was attributed to corticosteroids when administered orally, by intramuscular route or in combination with antihistamines, whereas topical antihistamines were also considered well tolerated but with reduced efficacy (Table 2).

### Data on Patients Population

A total of 2778 patients were included in the participating countries. Egypt, Mexico and Brazil included the higher number (45%). The remaining was equally distributed in Colombia, Guatemala, Iran, Venezuela, Argentina, Israel, Kuwait and UAE. 2776 met inclusion criteria and were included in the analysis.

Patients’ characteristics are described in Table 3.

**Table 3:** **Patients’ demographics and characteristics (N = 2776)**

**Age, median (range)**	**29 [5–94]**
**Gender, female n (%)**	1510 (54.4)
**Ethnicity**	**n (%)**
Native Latin-America	888 (32.0)
Caucasian	863 (31.1)
Oriental, Arab, Persian	791 (28.5)
Asian	99 (3.6)
Others	135 (5.93)
**Place of residence**	**n (%)**
Urban	2390 (86.1)
Suburban	182 (6.6)
Rural	138 (5.1)
**Country**	**n**
Egypt	500
Mexico	418
Brazil	351
Colombia	223
Guatemala	216
Iran	207
Venezuela	201
Argentina	200
Israel	176
Kuwait	150
UAE	134
**Occupation**	**n (%)**
Professional	693 (25.0)
Housewife	334 (12.0)
Self-employed	164 (5.9)
Skilled labor	141 (5.1)
General labor	110 (4.0)
Business	101 (3.6)
Retired	93 (3.4)
Farmer	10 (0.4)
Disability	10 (0.4)
Other	1071 (38.6)
Missing	49 (1.8)
**Smoking status, n (%)**	
Never	2265 (81.6)
Former	286 (10.3)
Current	225 (8.1)
**Medical History, n (%)**	
Respiratory disease	1219 (43.9)
Allergy	1315 (47.4)
ENT disease	1292 (46.5)

The median age of the included population was 29 years old (range 5–94 years) with males and females equally distributed. They were Latin-Americans (32%), Caucasians (31.1%), Middle-oriental (28.5%), Asian (3.6%) and, others 135 (5.93). Concerning working activity at the time of the survey, most patients were active (25%) and housewives (12%). Considering that about 40% (mainly in the 668 patients under 18 years old) declared other unspecified occupation, remaining people were self-employed, skilled labor, general labor, in business affairs, in pension, or affected by disability and under social security allowance. They were mainly urban area residents (93%).

Concerning the patients’ smoking status, 81.6% had never smoked, 10.3% were former smokers and 8.1% were current smokers. History of any respiratory disease in the course of their life was reported by 43.9% of patients; in particular 47.4% had a history of allergy and 46.5% of ENT conditions (Table 3).

### Data on disease characteristics

In this survey, the ARIA classification based on the duration and impact of the disease was the dominant approach in clinical practice, with persistent and intermittent rhinitis identified in 33.4% and 30.7% of cases respectively. However, the previous classification based on symptoms seasonality appeared still adopted: seasonal allergic rhinitis was diagnosed in 27% and perennial allergic rhinitis in 15.1%. The severity of symptoms was mild to moderate in a high percentage (45.4% and 37.3% respectively); only 12.9% of patients judged them as severe. Regarding frequency, most symptoms were present less than 4 days a week (31%). Patients suffering more than 4 days a week were 25.3%. Those suffering more or less than 4 consecutive weeks were 23.3% and 16.4%, respectively (Table 4).

**Table 4:** **Disease characteristics**

**Type of rhinitis, n (%)**	Persistent 926 (33.4)			Intermittent 853 (30.7)	
	Seasonal 748 (27.0)			Perennial 419 (15.1)	
**Key Rhinitis Symptoms for Physician Diagnosis, n (%) **	Nasal congestion 2355 (84.8)			Sneezing 2195 (79.1)	
	Rhinorrhea 2106 (75.9)			Itching 1935 (69.7)	
**Main nasal symptom according to patients, n (%)**	Nasal blockage 2331 (84)			Sneezing 2221 (80)	
	Nasal discharge 2101 (76)			Itching 1855 (67)	
**Intensity of symptoms according to patients, n (%)**	Severe 357 (12.9)			Moderate 1261 (45.4)	
	Mild 1035 (37.3)			Absent 123 (4.4)	
**Frequency of symptoms, n (%)** ^**£**^	<4 days/week: 850 (30.6%)			>4 days/week: 702 (25.3%)	
	<4 consecutive weeks: 456 (16.4%)			>4 consecutive weeks: 646 (23.3)	
**Causes of symptoms according to patients, n (%)**	Outdoor:			Non-specific:	
	-pollens 1126 (40.6)			- climate changes 2252 (81.1)	
	Indoor:			- irritants/pollutants 1422 (51.2)	
	- mites 2339 (84.3)			- infections 775 (27.9)	
	- moulds 905 (32.6)			Others:	
	- dander 851 (30.7)			- food 511 (18.4)	
				- drugs 227 (8.2)	
				- latex 168 (6.1)	
**Sensitizations documented by skin test, n (%)**	House dust mites 892 (82.9)			Pollens 506 (47%)	
	Moulds 312 (29.0)			Other 253 (23.5)	
	Pets dander 301 (28.0)				
**Co-morbidities, n (%)**	Sinusitis 1384 (49.9)			Otitis media 363 (13.1)	
	Asthma 907 (32.7)			Nasal polyps 310 (11.2)	
	Conjunctivitis 1005 (36.2)				
**Respiratory symptoms within the last 12 months according to patients, n (%)**		Cough	Wheezing	Dyspnea	Chest tightness
	Recurrent	1244 (44.8)	764 (27.5)	857 (30.9)	535 (19.3)
	Nocturnal	1084 (39.1)	695 (25.0)	783 (28.2)	436 (15.7)
	Post-exercise	894 (32.2)	731 (26.3)	954 (34.4)	630 (22.7)

^£^Missing data: 122 (4.39%).

Nasal congestion was the most bothersome symptom motivating the physician visits (84.8%), followed by sneezing (79.1%), anterior rhinorrhea (75.9%) and nasal itching (69.7%).

Patients attributed the onset of symptoms to indoor house dust mites exposure in 84.3% of cases, but also to molds (32.6%), pet dander (30.7%) and pollens were identified as possible causes among outdoor exposure in 40.6% of cases. Other possible allergens were food (18.4%), drugs (8.2%) and latex (6.1%), and not specific factors, like climate changes (81.1%), pollutants (51.2%) and infections (27.9%).

**Figure 3:**
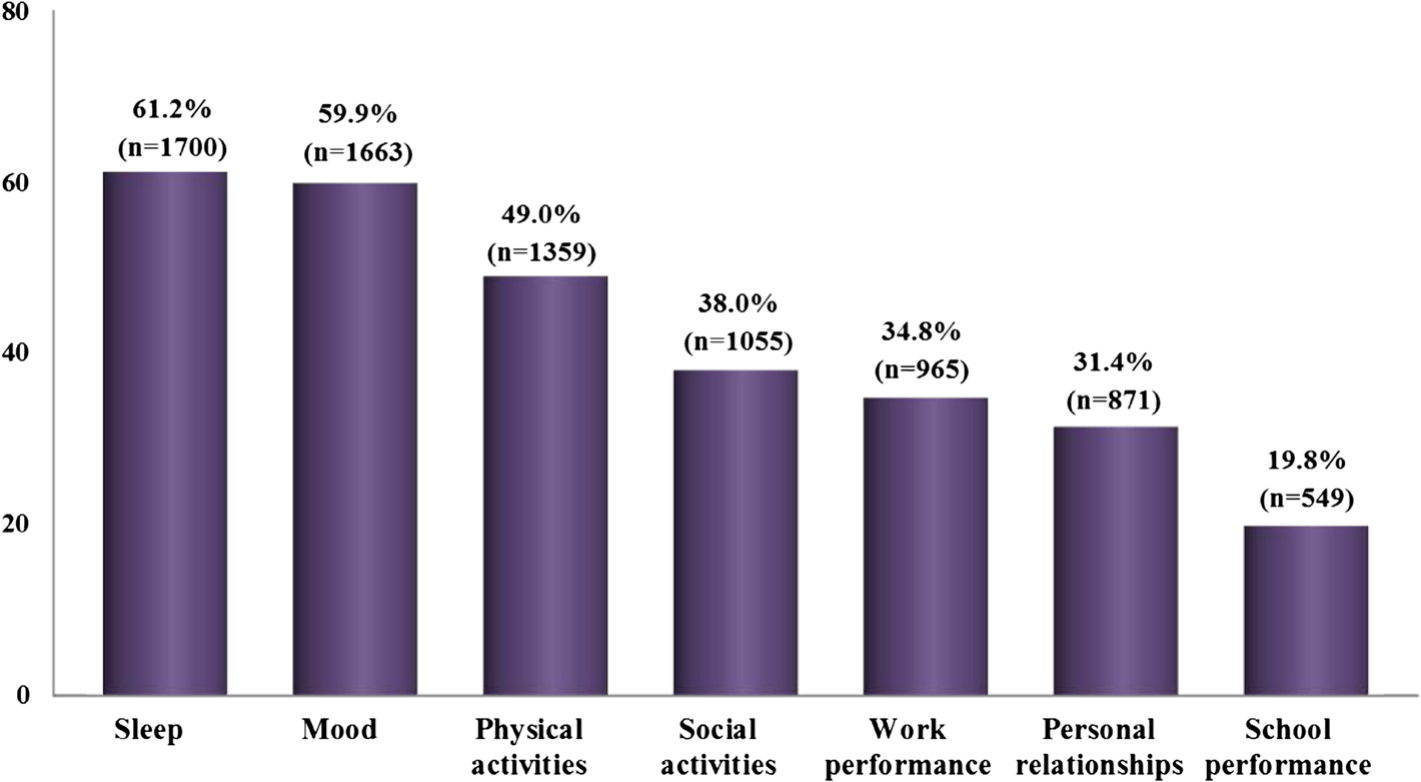
**Impact of rhinitis on quality of life [Number and percentage of patients].** Impairment caused by rhinitis.

Patients’ QoL, sleep, mood and physical activities appeared particularly altered in 61.2%, 59.9%, and 49% of cases, respectively), followed by social activities (38%), labor (34.8%), school (19.8%) performances and, personal relationships (31.4%) (Figure 3).

Sinusitis and conjunctivitis were frequent past or current co-morbidities in (49.9%) and (36.2%) of patients respectively. The coexistence of otitis media (13.1%) and nasal polyposis (11.2%) was less frequent.

Asthma was present in 32.7% and hospitalization for exacerbations within the last 12 months was reported by 12.9% of patients. When surveyed on the frequency of respiratory symptoms in the last year, 44.8% of patients described cough as recurrent, 39.1% as nocturnal and 32.2% following exercise. For wheezing the percentages were 27.5%, 25% and 26.3% respectively. Dyspnea occurred recurrently in 30.9% of patients, during the night in 28.2% and after exercise in 34.4%. Chest tightness was the less perceived symptom, occurring mainly as exercise-induced (22.7%), recurrently in 19.3%, and during the night in 15.2%.

The median number of years before rhinitis diagnosis was 6, ranging from 1 up to 62. Patients reported that the clinical diagnosis was frequently confirmed by additional procedures, like physical examination (95%), radiology (45%) and CT-scan (29%). Among nasal tests, endoscopy was frequently used (27.6%), followed by nasal cytology, biopsy or nasal culture (8.4%), nasal air flow measurement (2.3%), allergen specific nasal challenge (1.6%) and mucociliar test (0.7%). Information regarding the allergic status was obtained from skin tests (38.8%), serum eosinophilia (22.6%), total (21.1%) and specific (5.2%) serum IgE levels.

Skin tests were positive to house dust mites in 82.9% from 1076 patients (38.8% of the whole population), followed by pollens (47%), molds (29%), animal dander (28%), or other allergens (23.5%). Data on disease characteristics are summarized in Table 4.

### Data from patients’ on disease management

**Figure 4:**
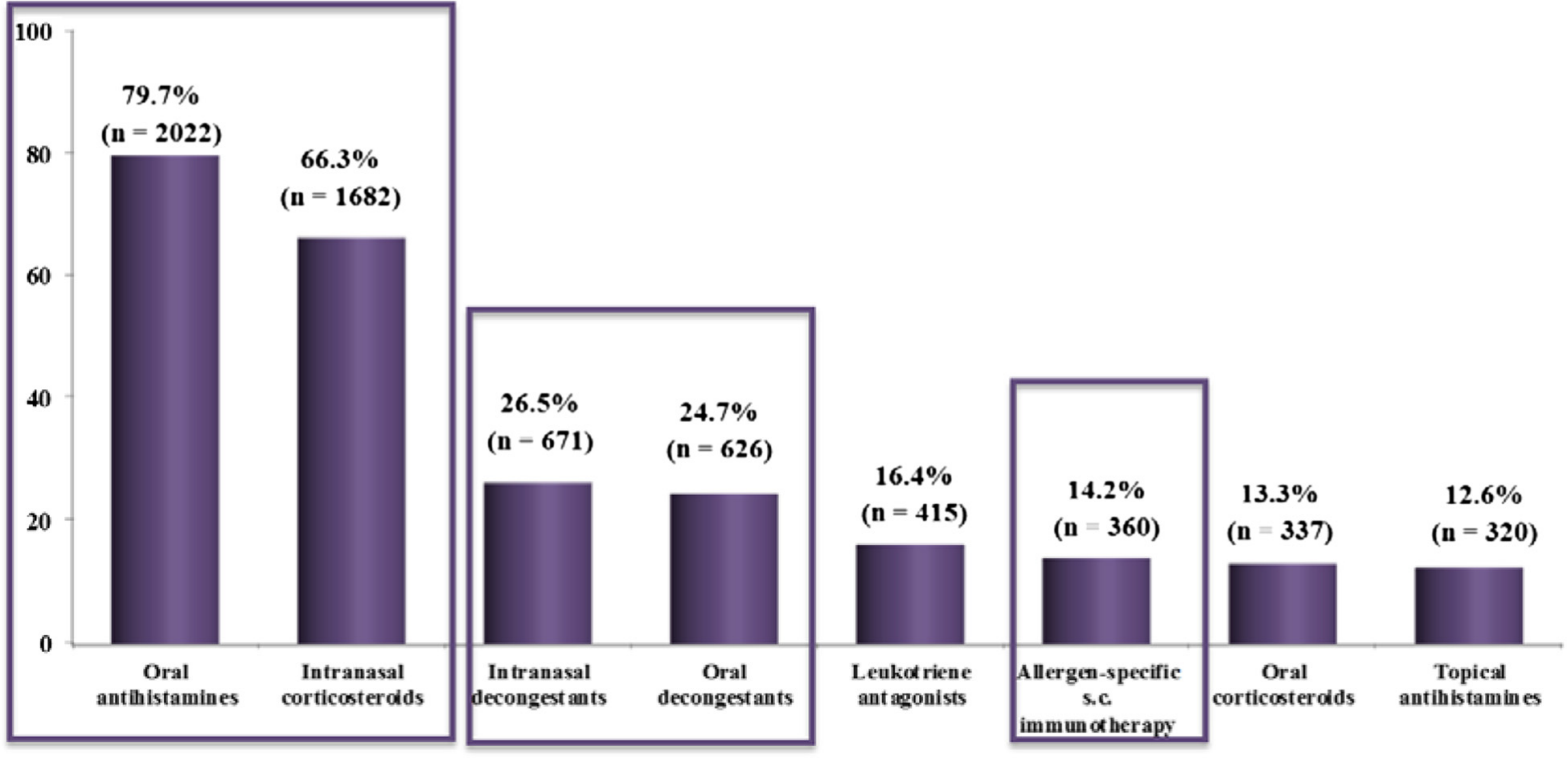
**Current treatments for rhinitis [Number and percentage of patients].** 2537 patients received at least one treatment. Only percentages superior to 12 are mentioned. s.c.: subcutaneous.

Out of 2776 patients, 93.4% (2592) had somehow received a recommendation to avoid allergens and irritant agent exposure. Notably, 2537 (91.4%) were receiving at least one treatment at the time of the survey, mostly oral antihistamines (79.7%) and intranasal corticosteroids (66.3%) (Figure 4).

Less prescribed were oral (24.7%) or topical decongestants (26.5%), leukotriene antagonists (16.4%), allergen specific subcutaneous immunotherapy (14.2%), oral steroids (13.3%), topical antihistamines (12.6%) or sublingual immunotherapy (3.1).

**Figure 5:**
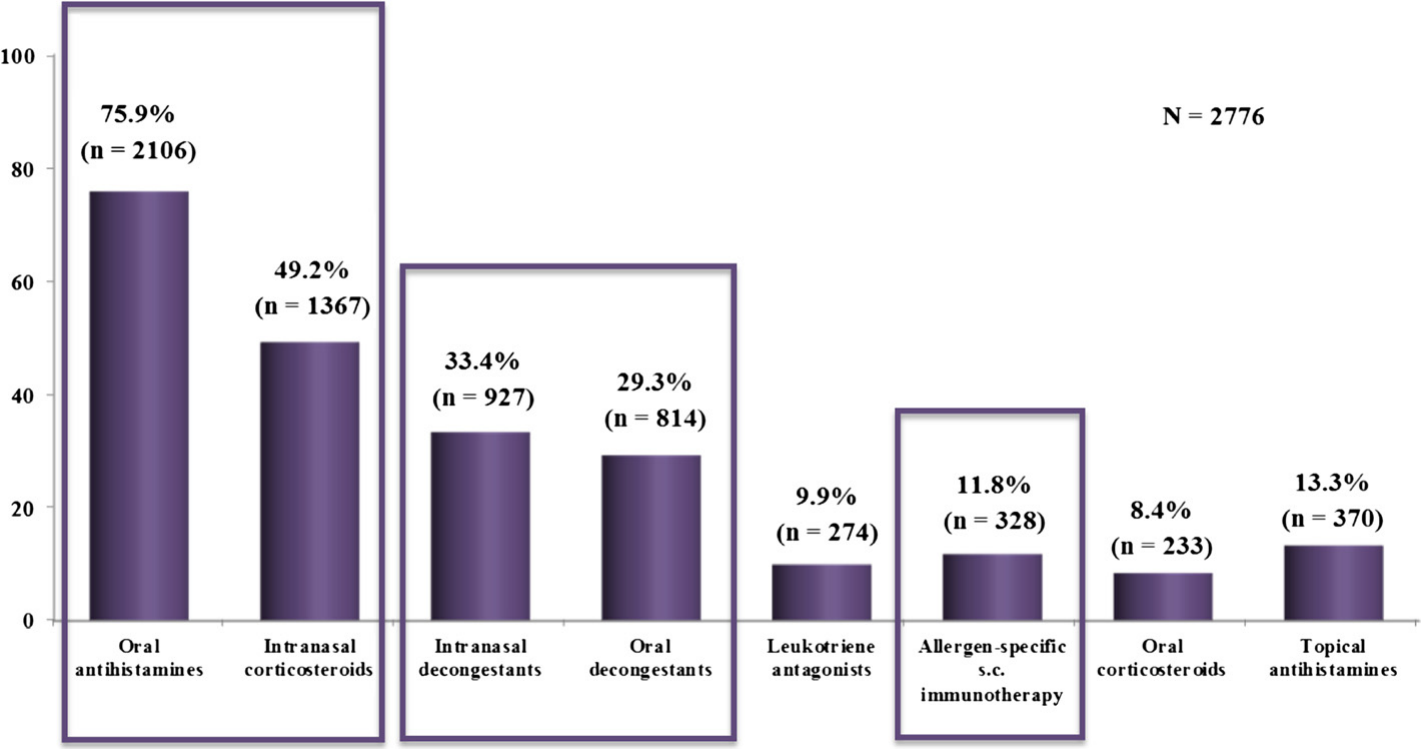
**Patients’ preferences regarding treatment for rhinitis [Number and percentage of patients].** Only percentages superior to 8 are mentioned. s.c.: subcutaneous.

Oral antihistamines and topical steroids were preferred by 75.9% and 49.2% of patients, respectively, followed by topical (33.4%) and oral (29.3%) decongestants, topical antihistamines (13.3%), allergen immunotherapy (11.8%), leukotriene antagonists (9.9%), and oral steroids (8.4%). Oral and intranasal routes of administration (60.3% and 32.4%, respectively) were preferred to the injectable (6.2%) or others administration routes (1.1%) (Figure 5).

When patients’ and doctors’ judgments on treatment compliance were compared, different viewpoints leap out. According to patients, compliance was excellent (31.2%), very good (29.7%) and good (22.8%), which was different from the physicians’ point of view (7.7%, 37.2% and 41.9%, respectively). Very few patients (from 3 to 10%) judged it as poor or unsatisfactory (Table 4).

A different weight was attributed by the two counterparts to the factors affecting the treatment compliance. According to physicians, the drugs cost was the most important conditioning factor (70.5%), followed by the fear of reported adverse events (45.7%), the need for frequent doses (38.9%), the efficacy of ongoing treatment (35.9%), the occurrence of drug-related adverse events (32.5%) and the route of administration (25.2%). Conversely, patients referred to be mainly bothered by the need of frequent doses (33.7%) and the cost of medications ranked in second position (32.9%). Patient did not appear particularly worried by the fear of reported (18.1%) or drug related (19.5%) adverse events. On the other hand, some patients considered the current ongoing therapy efficacy (24.8%) and the route of administration (19.3%) as a cause affecting the compliance (Table 4).

Finally, when patients were asked if they have received any kind of education for the management of their condition, a written set of indications was referred to by 51.4% of them. Most patients received oral explanations about the characteristics of their disease (85.2%) and its treatment (80.6%); only 58.1% received information about side effects or other aspects (21.1%).

### Peculiarities in different areas

The included population mean age ranked between 22 and 27 years old in all participant countries.

No significant differences were observed in terms of management and guidelines implementation between the different Latin-American countries. Differences were observed in the frequency of the different comorbidities, the frequency of sinusitis was higher in Argentina (62%) and Guatemala (57%), compared to the other countries. The frequency of asthma varied between 28% in Mexico and 46% in Brazil. In addition, the use of skin tests to detect the main allergen provoking the rhinitis varied between the countries and ranks from 24 to 60%.

In Middle–East countries, no significant differences were observed in terms of management even if the implementation of guidelines was lower than in Latin American countries.

The use of skin testing to identify the type of allergen was very low compared to the Latin American countries ranging from 9 to 29%. In terms of comorbidities, the frequency of sinusitis ranged from 34% in Israel to 61% in Iran. Asthma frequency appeared lower than in Latin America varying between 18% in UAE to 37% in Egypt.

## Discussion

Designed according to the most accepted epidemiological recommendations, ISMAR was aimed to be the first-ever global, quantitative survey to ask separate groups of patients and physicians similar questions to identify differences in attitudes and opinions on the management of AR.

A high percentage of physicians appeared acknowledged about the existence of the most common international guidelines, recognizing their importance in conditioning an optimal disease management. Current guidelines point out the importance of patients’ reported outcomes in decision making. Despite that, the suggestion of guidelines in evaluating patients’ QoL seemed poorly followed, with an apparent low trust in their usefulness for treatment decisions. However, this is probably due to the time consumed in filling this type of questionnaire in a real clinical life. Some physicians paid attention to sleep disturbance provoked by AR.

Concerning the diagnostic approach, the clinical history appeared the most common criteria in categorizing patients on the basis of symptoms severity. The use of other diagnostic instruments, like questionnaires and visual analogue scales appears as unsatisfactory. The number of patients undergoing allergy testing after a diagnosis of nasal complaint was low (38.3%), probably affecting an optimal framing of the respiratory condition. This may be one the reasons for the observed low use of allergen-specific immunotherapy, despite the existing clinical evidence, as well as the no availability of allergen extracts in all participating countries.

As expected house dust mites and pollens were the most common causes of sensitization.

Pharmacotherapy recommendations of guidelines were generally implemented. Most of patients were already receiving treatment at the moment of the visit, mainly oral antihistamines, intranasal corticosteroids and decongestants, which were considered safe and effective by both, patients and physicians, particularly preferred through oral and nasal route of administration.

The typical patient from the surveyed countries referring to medical assessment with a nasal complaint was a non-smoker subject with mild to moderate persistent symptoms, mainly bothered by nasal congestion. This patient is likely able to interpret the causative factors and mentions a significant impairment of his/her QoL, social activities, work or school performances. As expected, patients presented comorbidities, mainly sinusitis or asthma.

A similar survey conducted in the USA among 447 patients with AR visiting their specialist or primary care physician for routine clinical care, found that a significant proportion of patients had moderate or severe disease (62.6%), persistent symptoms (47.6%) and comorbidities such as, asthma (28.8%) or sinusitis (12.5%). The results of this survey highlight the unmet needs of the many patients in the USA with moderate or severe and/or persistent disease and an associated high symptom burden and impaired health-related quality of life [13].

A cross-sectional study determining the spreading level of the WHO-ARIA (World Health Organization’s Allergic Rhinitis and its Impact on Asthma) guidelines and their influence on medical practices was conducted in France among 943 general practitioners (GPs) and 277 ENT [14]. About 54.4% of the physicians claimed to know the WHO-ARIA guidelines and 49.7% said they followed them. These results vary significantly, mainly according to medical specialty (ENT vs. GP). In comparison to those who did not know the guidelines, their patients benefited more frequently (P < 0.0001) from allergen search (42.2% vs. 31.7%), a nasal endoscopy (38.3% vs. 26.0%), a follow-up consultation (64.9% vs. 52.6%) and written information on rhinitis (30.7% vs. 14.1%). Paradoxically, they do not search more frequently for asthma and do not provide different first-line treatment strategy and duration [14].

The burden of allergic rhinitis was evaluated among patients from the members of European allergy patient organizations. The Patient Voice Allergy Survey was a quantitative, self-completion survey of 3562 patients with AR (16 years and older). Background information on AR, severity of AR symptoms and their impact on lives, nonmedical measures for relieving of symptoms, types of medications, and concomitant conditions were evaluated [15].

Almost 50% of the responders reported symptoms lasting for more than a season. Preventive household adjustments were considered as expensive and with little perceived benefit. Sleep and emotional life were affected by AR. Most patients were satisfied with the current AR medications; at least one-fifth reported dissatisfaction. Patients perceived that AR worsens other concomitant allergic diseases [15].

The classification of AR proposed by ARIA, based on symptoms duration and impact on patient’s quality of life and sleep, appeared widely adopted, although some physicians, in our study, probably preferred the previous one based on seasonality. Patients with disturbing symptoms visited the specialist 6 years after the onset of symptoms. This is surely an aspect worthy of improvement, together with the opportunity to educate doctors toward the evaluation of patients with nasal symptoms by means of allergen search.

Room for improvement is also suggested regarding treatment compliance, despite that patients’ judgments appear more optimistic in respect to physicians’. However, they are in agreement that the main causes of non-compliance are the cost of medication, the frequency of doses, the fear of adverse events and the poor efficacy. These should be points to be addressed in order to enhance the treatment compliance and the outcomes.

Encouraging information concerns the data on patients’ education. Most patients have received recommendations about the avoidance of allergen and triggers exposition, together with written or oral advices about the characteristics of their condition and its treatment. These aspects suggest that, overall, guidelines appear well known and useful to physicians and physician-patient communication is quite satisfactory.

Another international cross-sectional survey evaluated patient and physician perceptions of the effectiveness of treatment, symptoms, and the impact of AR. Out of 88 patients recruited in Spain, by primary care physicians and specialists, 77 (87.5%) had AR confirmed by symptoms and skin prick testing, measurement of specific immunoglobulin E, or nasal allergen challenge. Most patients had moderate or severe disease (67.0%), which was assessed in terms of severity and persistence of symptoms, and comorbid conditions such as asthma and anxiety. Nasal and ocular symptoms were reported by 83% of patients, either currently or frequently, and 36.4% of patients reported that these symptoms were moderate or severe. More than half of the patients (59.1%) were using 2 or more medicines to manage their AR, and 73.7% of patients taking a non-sedating antihistamine plus an intranasal corticosteroid had moderate or severe disease. Most patients (83.1%) reported some impact from the symptoms of AR on daily activities. The mean (SD) mini RQLQ score was 2.4 (1.4) in patients with mild disease, 2.6 (1.2) in patients with moderate disease, and 3.3 (2.3) in patients with severe disease. In this survey physicians estimated that only a minority of patients had symptoms that were poorly controlled, more than one-third of patients reported that their nasal and ocular symptoms were moderate or severe in nature, and most patients considered that their symptoms had an impact on their daily activities, work/school performance, and sleep patterns. The authors concluded that these differences highlight the need for more objective discussion between patients and physicians on the nature, severity, and impact of symptoms, as well as treatment approaches, and how to obtain maximum benefit from currently available prescription medications [16].

## Conclusion

In conclusion, the ISMAR registry provided interesting information regarding the management of rhinitis from a patient and physician points of view as well as on the knowledge of guidelines for an optimal management with many similarities between the participating countries.

Further efforts are required to better manage AR and its comorbidities.
